# Neutropenic Sepsis in the ICU: Outcome Predictors in a Two-Phase Model and Microbiology Findings

**DOI:** 10.1155/2016/8137850

**Published:** 2016-04-18

**Authors:** Jan M. Kruse, Thomas Jenning, Sibylle Rademacher, Renate Arnold, Clemens A. Schmitt, Achim Jörres, Philipp Enghard, Michael Oppert

**Affiliations:** ^1^Klinik mit Schwerpunkt Nephrologie und Internistische Intensivmedizin, Charité Universitätsmedizin Berlin, Germany; ^2^Klinik mit Schwerpunkt Hämatologie und Onkologie, Charité Universitätsmedizin Berlin, Germany; ^3^Abteilung für Notfall- und Intensivmedizin, Klinikum Ernst von Bergmann Potsdam, Germany

## Abstract

*Objective*. Patients with neutropenic sepsis have a poor prognosis. We aimed to identify outcome predictors and generate hypotheses how the care for these patients may be improved.* Methods*. All 12.352 patients admitted between 2006 and 2011 to the medical ICUs of our tertiary university center were screened for neutropenia; out of 558 patients identified, 102 fulfilled the inclusion criteria and were analyzed. Severity markers and outcome predictors were assessed.* Results*. The overall ICU mortality was 54.9%. The severity of sepsis and the number of organ failures predicted survival of the primary septic episode (APACHE II 22.8 and 29.0; SOFA 7.3 and 10.1, resp.). In the recovery phase, persistent organ damage and higher persistent C-reactive protein levels were associated with a poor outcome. Blood transfusions and CMV infection correlated with an unfavorable prognosis. Ineffective initial antibiotic therapy, fungal infections, and detection of multiresistant bacteria displayed a particularly poor outcome. Infections with coagulase-negative staphylococci and enterococci were associated with a significantly higher mortality and a high degree of systemic inflammation.* Conclusion*. Patients with persistent organ dysfunction show an increased mortality in the further course of their ICU stay. Early antimicrobial treatment of Gram-positive cocci may improve the outcome of these patients.

## 1. Background

Many intensivists and oncologist share the often frustrating experience of caring for patients with neutropenic sepsis: infections are a frequent complication in leukopenic patients, affecting an estimated 24% patients after chemotherapy for hematologic diseases or solid organ tumors [1]. Although in many cases the underlying disease is potentially curable, once having to be transferred to the ICU with sepsis these patients have a poor prognosis.

In former decades, the prognosis of these patients was regarded as so poor that it was debated whether to admit them to the ICU at all [2–5]. Subsequently, several studies tried to identify prognostic markers for the outcome of these patients, intending to help guiding the decision which patients to admit to the ICU [6–8].

Potentially due to improved oncologic and intensive care treatment regimes, recent studies demonstrated a reasonable outcome of patients with neutropenic sepsis in the ICU, documenting hospital survival rates of 50–60% [6, 9, 10]. Further data suggest that long-term prognosis of cancer patients treated in the ICU does not differ from that of patients who do not need ICU treatment [11, 12]. In line with these reports today the debate whether to generally deny these patients admission seems resolved and they are routinely treated in ICUs.

Nevertheless, current studies addressing the prognosis of patients with neutropenic sepsis in the ICU are scarce, and more importantly, there are only few studies aiming to identify strategies how to improve the outcome of these patients. Particularly little is known about the impact of the infecting organism. Furthermore, it is unknown what determines ICU mortality of patients who survive the initial septic episode but remain dependent on intensive care medicine. Here we present our results of a retrospective cohort study aiming to clarify circumstances influencing the course of the ICU-stay, determining ICU mortality, and how the infecting organisms impact outcome in these patients.

## 2. Methods

### 2.1. Patients

Neutropenic patients admitted to the medical intensive care units of the Charité-University Medical Center, Campus Virchow (CVK), Berlin, a university tertiary care center, meeting the criteria of sepsis in the years 2006 to 2011, were included in the study. The study has been approved by the ethics committee of the Charité-University Medical Center. According to the decision of the ethics committee, no consent from the patients was needed due to the retrospective character of the study. The study was conducted according to the principles of the Declaration of Helsinki (World Medical Association 2008).

### 2.2. Antibiotic Regimen

Due to the retrospective nature of the study, the choice of antibiotics was made by the intensivist in charge. Standard operating procedures in our institution are based on international guidelines like those published by the IDSA. Depending on the suspected focus and preexisting or emerging organ system different regimens may be installed.

### 2.3. Data Collection

Data were collected from the electronic patient files. Neutropenia was defined as a neutrophil count of <500/mm^3^ or a leukocyte count of <1000/mm^3^ in the first 96 hours after ICU admission. Neutropenia had to last for at least 24 hours confirmed by two independent blood samples. Patients had to be treated for at least 24 hours on the intensive care unit to be included in the study. Sepsis was defined in accordance to the consensus definition by the Surviving Sepsis Campaign (SSC) guidelines [13]. Number of blood transfusions, length of mechanical ventilation, and extracorporeal renal support were also documented. Focus of infection as diagnosed by the intensivist in charge and results of microbiological cultures were registered. Coagulase-negative staphylococci and enterococci were only classified as infecting organism if present in more than one blood culture. Culture results were regarded as relevant for the initial sepsis when they were acquired up to 7 days before or after ICU admission. Fungal infection was classified following the European Organization for Research and Treatment of Cancer (EORTC) guidelines [14]. Results of virology examinations were also documented. Respiratory failure was defined as intubation in the ICU, tracheotomy, or noninvasive ventilation for at least 3 h per day. Circulatory failure was defined by the need of vasopressors as prescribed by the intensivist in charge. Liver failure was defined by bilirubin levels > 2 mg/dL or an international normalized ratio (INR) > 2 or a prothrombin time < 50. Kidney failure was defined according to the RIFLE classification or requirement of renal replacement therapy [15]. Sequential organ failure assessment score (SOFA) and Acute Physiology and Chronic Health Evaluation Score II (APACHE II) were documented at admission and at the time point of overcoming the sepsis as were the different parameters for organ dysfunction. The ICU stay was divided into two phases. The initial septic crisis and, if it was survived, a second phase until discharge from the ICU. The initial sepsis was considered resolved when the patient was free of vasopressors, extubated, or required less than 3 h of noninvasive ventilation per day, had a decrease of C-reactive protein (CRP) or procalcitonin of more than 50% for more than 48 h, or no systemic inflammatory response syndrome (SIRS) criteria for more than 24 h.

Our intention was to identify factors that predicted ICU mortality if the initial crisis was overcome. 558 patients with neutropenia were screened and 102 meeting the aforementioned sepsis criteria were included.

### 2.4. Statistical Analysis

Parameters were recorded as median and interquartile range (25th–75th percentile). Categorical variables were calculated using the chi-square test or Fisher's exact test. Continuous variables were calculated using the Mann-Whitney *U* test. Odds ratios were calculated using binary logistic regression.

SPSS (Version 19, IBM) was used and graphs were generated using GraphPad prism 5.0.

## 3. Results

### 3.1. General Patient Characteristics

**Figure 1:**
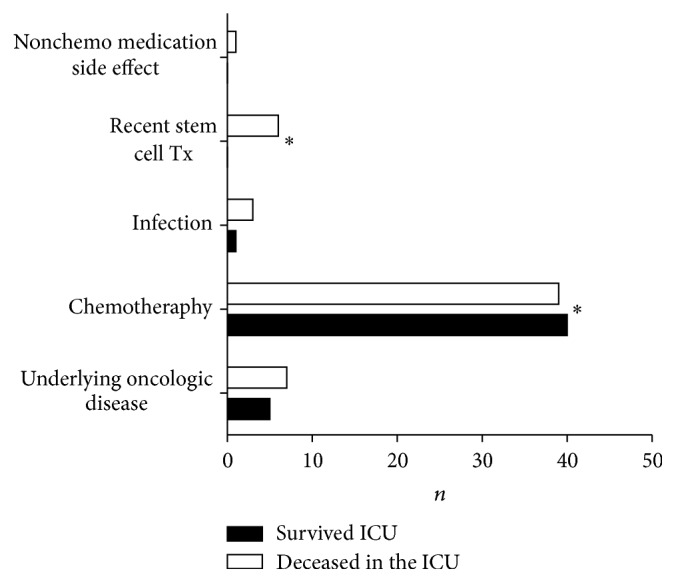
Context-dependent mortality of leukopenic patients admitted to the ICU. Patients after stem cell transplantation have an inferior prognosis. Patients with leukopenia after chemotherapy have a significantly better ICU survival. *∗* refers to statistical significance with *p* < 0.05.

Among the 12.352 patients admitted to our ICU ward between 2006 and 2011, 558 neutropenic patients were identified and 102 patients fulfilling the criteria for neutropenic sepsis were included in our analysis. Leukopenia was attributable to chemotherapy (*n* = 79), underlying hematologic disease (*n* = 12), infection (*n* = 4), side effect of nonchemotherapeutic drugs (*n* = 1), autologous bone marrow transplantation (*n* = 2), and allogenic stem cell transplantation (*n* = 4). The six patients after stem cell transplantation all had a recent transplantation, 8–28 days (median 10, 5 d) prior to referral to the ICU. One further patient after allogenic stem cell transplantation was included in the study; however, the transplantation was not recent and the leucopenia was attributable to infection. Overall ICU mortality was 54.9% and 28 day mortality was 52.0%; all patients with stem cell transplantation (*n* = 6) deceased during their ICU stay (Figure 1).

### 3.2. Model of Two Phases of the ICU Stay

**Figure 2:**
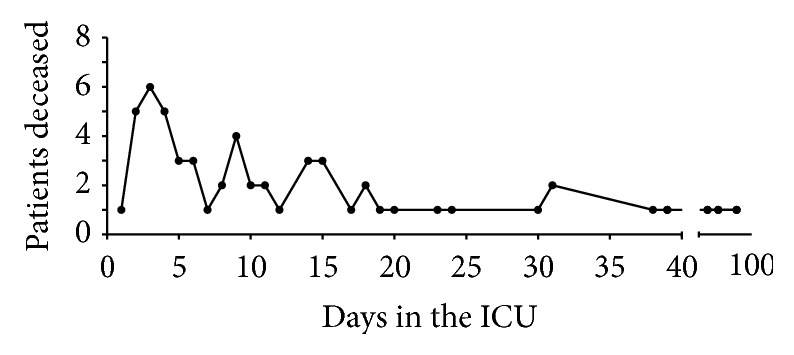
Number of patients deceased per ICU day. There is a concentration of fatalities in the first 6 days.

**Figure 3:**
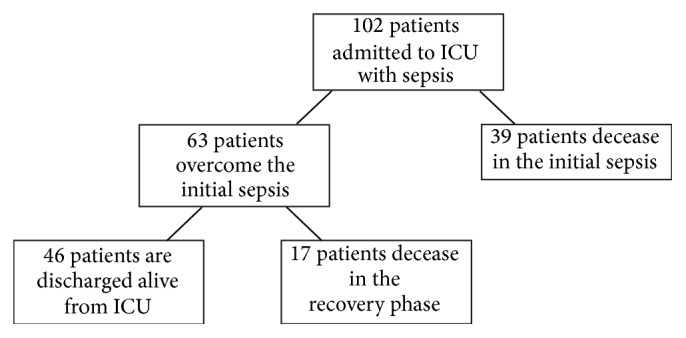
Overview of the number of analyzed patients. Of the 102 patients admitted to the ICU, 39 patients deceased in the initial sepsis phase, while 63 patients overcame it. Of these 63 patients, 17 deceased in the ICU in the recovery phase, while 46 patients survived and were discharged from the ICU.

In the first days after admission to the ICU, we observed a particularly high mortality burden, with 22.5% of all admitted patients deceasing in the first 6 days. The other fatalities occurred over a more prolonged period of time (Figure 2). In order to analyze which patient characteristics are predictive of overcoming the initial sepsis and which characteristics determine whether the patient survives the entire ICU stay, we divided all ICU stays in potentially two phases: First the initial septic episode, followed by a recovery phase if the patient survived the initial crisis. As defined by our criteria for overcoming the initial sepsis, out of the total 102 patients 63 survived the initial septic crisis, while 39 patients failed to overcome the initial sepsis and deceased during that phase. Of the 63 patients that were successfully treated for the initial sepsis, 17 patients consecutively died during the recovery phase, while 46 patients were discharged alive from the ICU (Figure 3).

### 3.3. Severity of Sepsis and Organ Failure Predicts Overcoming or Not of the Initial Sepsis

The patient group that did not overcome the initial sepsis episode was admitted to the ICU with a significantly higher respiratory rate, higher first documented serum lactate value and higher APACHE II and SOFA scores as compared to the group that survived the initial sepsis. Patients that failed to overcome the initial crisis were more likely to be intubated and had a lower oxygenation index. A similar proportion of both groups received vasopressors on admission, and there was no significant difference in the mean arterial pressure (MAP), but the patients who did not overcome the first sepsis episode received significantly more fluids in the first 24 h and had a higher maximal noradrenaline dose. They more often presented liver and renal failure, and the group with persistent sepsis received more transfusions of erythrocytes, platelets, and fresh frozen plasma. There was no difference in the highest CRP as a marker for inflammation; however, the patients who overcame the sepsis achieved lower CRP values. Furthermore, longer duration of leukopenia prior to ICU admission was associated with worse outcome (Table 1).

### 3.4. Persistent Organ Damage and High Initial CRP Predicts Poor Outcome after Overcoming the First Sepsis

There was no significant difference in the disease severity on admission to the ICU between those patients who survived the initial sepsis phase and those who died later on despite overcoming the initial sepsis. Both groups had similar respiratory rates, first documented serum lactate values, APACHE II and SOFA scores when admitted to the ICU. There was no difference in the MAP, fluids administered, or need for vasopressors in the first 24 h. The group of nonsurvivors had a higher frequency of circulatory failure after overcoming the initial sepsis and a higher maximal noradrenaline dose in their total ICU stay. The nonsurvivors where more often intubated, underwent tracheotomy more frequently, and showed persistent lung failure after the first sepsis episode. Likewise, renal and liver failure was more frequent in the group with the unfavorable outcome. The nonsurvivors received more erythrocyte, platelet, and fresh frozen plasma transfusions per ICU day. Furthermore the nonsurvivors had a significantly higher CRP in the first 10 days, which persisted in the course of their ICU stay, indicating a higher initial and maintained extent of inflammation (Table 2).

### 3.5. Impact of Microbiologic Findings on the ICU Outcome

**Figure 4:**
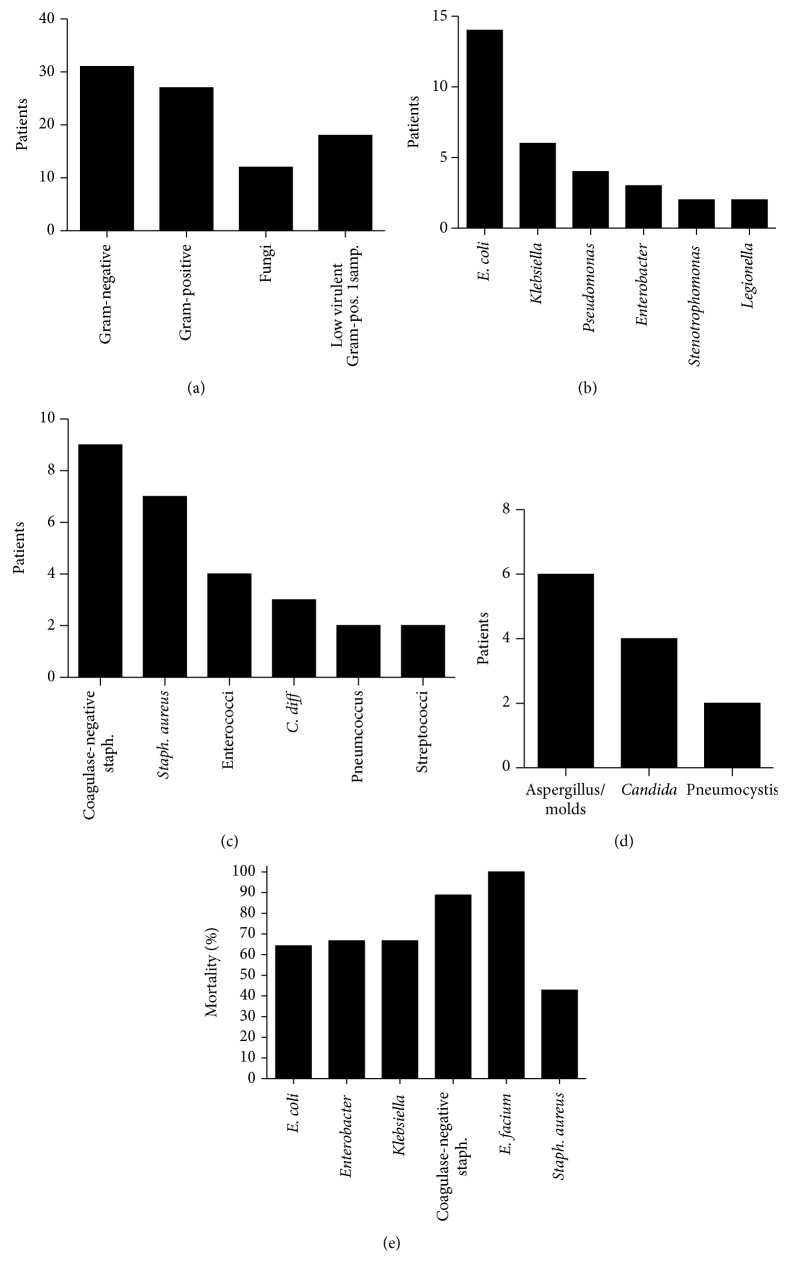
Microbiologic findings in the initial sepsis phase. (a) Overview of the pathogens identified in the initial sepsis. (b) The most commonly identified Gram-negative bacteria (displayed are the bacteria that were observed in at least 2 patients). (c) The most frequently found Gram-positive bacteria (displayed are the bacteria that were observed in at least 2 patients). (d) Fungi observed in the microbiologic diagnostic. (e) The mortality of the 6 most frequently found bacteria.

In 65 patients, the microbiological testing identified bacteria and/or fungus associated with the initial sepsis. The identified species were Gram-negative bacteria in 31, Gram-positive bacteria in 27 patients, and in 12 patients, microbiology findings indicated a fungal infection. Since coagulase-negative staphylococci and enterococci are frequent contamination of blood cultures [16, 17], positive cultures with these bacteria were only considered indicative of an infection if present in at least two cultures; single positive cultures were regarded as suspicious of contamination instead of infection and are displayed separately (Figure 4). The most common Gram-negative microorganism was* E. coli*, and the most frequent Gram-positive were coagulase-negative staphylococci. Aspergillus and other molds were the most frequently observed fungal infections. Interestingly, among the six most frequently identified bacteria, coagulase-negative staphylococci and* Enterococcus faecium* were associated with a higher mortality (significantly higher mortality compared to all other patients with positive microbiologic culture results, *p* = 0.039) (Figure 4). Furthermore, patients who deceased in the ICU had significantly longer indwelling central venous lines than patients who survived (*p* = 0.009) (data not shown).

In 37 patients, no causative agent for the initial sepsis was identified. Overall, culture-positive patients had a worse prognosis than patients without successful identification (46.2% versus 24.3% failed to overcome the initial sepsis, with 66% versus 35.1% total ICU mortality). Patients with noneffective antibiotic therapy had an increased ICU mortality of 81.8% (*n* = 11). All patients with evidence of a fungal infection (4x Aspergillus antigen positive, 1x detection of aspergillus in airways, 1x aspergillus in tissue biopsy, 4x Candida species in blood culture, 2x* Pneumocystis jirovecii* in airways, and 1* Saccharomyces* species in blood culture) as cause of the initial sepsis deceased in the ICU (*n* = 13). Proof of infection or colonization with multiresistant bacteria during the total ICU treatment was associated with higher mortality (81.8%, *n* = 22).

A total of 39 patients were tested for active cytomegaly virus (CMV) replication during their ICU stay. Of these 39 patients, 6 had active CMV replication, and all 6 patients deceased during their ICU treatment (data not shown).

## 4. Discussion

In this retrospective cohort study, we examined patients with neutropenic sepsis admitted to a medical ICU. We report here total mortality and survival times at the ICU and assessed different predictors of ICU mortality in the different phases of the ICU stay. Additionally, we analyzed microbiologic findings and their association with the clinical course.

The overall ICU-mortality in our cohort was 54.9%. While significantly higher mortality rates were reported for neutropenic patients in former decades [18], Legrand et al. [10] recently presented a comparable mortality rate of 49.8% in their cohort of patients with neutropenic sepsis. A group of cancer patients with various diagnoses examined by Pené et al. [19] showed an ICU-mortality of 52.7%, which is also in the same range as ours, although not only septic patients were examined. Mokart et al. [20] found a surprisingly low ICU-mortality of 23% comparable to the numbers reported for infected patients without neutropenia, for example, in a large multicenter trial by Vincent in 2008 [21]. Potential differences in the study populations may account for the reported variability in survival, since factors such as the reason for neutropenia may exert a strong impact on outcome.

In our study, we chose to adopt a two-phase model to analyze our cohort of patients. While many patients died during the initial septic shock episode, a significant number of patients succumbed later after they had overcome the primary sepsis phase. We believe that it is more useful to assess these patient groups separately instead of analyzing them together, since the factors predicting outcome in the initial sepsis and the recovery phase may differ substantially.

Once the first sepsis is overcome, persistent organ failure is associated with poor survival, regardless of whether it was respiratory, circulatory, renal, or hepatic failure. Additionally, a high initial CRP and higher persistent CRP levels indicated poor outcome, stressing the importance of successful source control and adequate antibiotic therapy in early in the course by prescribing adequate antibiotic regimens and choosing adequate doses and taking other actions aiming at source control like replacement of central lines or removing them if possible. Another possible explanation might be that an elevated immune response in the patients with higher markers of inflammation may contribute to worse prognosis. Unlike the patients that were lost during the initial sepsis, the illness severity scores at admission had no impact on survival once the first sepsis episode was resolved. One might suggest that any organ failure remaining after successful therapy of the initial insult leaves the patient more susceptible to a second hit, most often secondary infection. A state of immune paralysis related to any failing organ system may be causally accused as well as prolonged immobilization or a state of persisting catabolic metabolism. Our data do not allow drawing a safe conclusion regarding a definite cause.

Duration of neutropenia is often considered a main factor determining the prognosis of patients with neutropenic sepsis [6, 22]. While duration of prior neutropenia was associated with an unfavorable outcome in our study, the persistence of neutropenia was not detected as a predictive factor once the initial sepsis was overcome. Possibly our study was underpowered to detect the impact of resolving neutropenia or the impact of leukocyte recovery is overplayed by other factors such as persistent need for organ support.

Unexpectedly, patients with a positive microbiological culture result showed a significantly higher mortality than those patients with negative results. To us the most reasonable explanation is that negative cultures point to an adequate selection of antibiotics prior to ICU-admission on the ward, since all patients were already under antibiotic treatment when admitted to the ICU. Another possible explanation is that culture-negative patients may have suffered from a SIRS of noninfectious origin, which might carry a better prognosis depending on etiology. Furthermore, it may be reasonable to assume that the likelihood of identifying a causative microorganism increases with extent and severity of the infection. Additionally, negative cultures after admission to the ICU may reflect successful coverage of the culprit organism by the antimicrobial treatment given prior to ICU admission. As expected, patients with an inadequate initial antimicrobial therapy had a significantly higher mortality, which is consistent with previous reports [23–25].

Interestingly, infections with coagulase-negative staphylococci (CNS) and* Enterococcus* spp. showed a higher degree of systemic inflammation mirrored in a significantly higher CRP level and a higher ICU mortality. In comparison,* Pseudomonas aeruginosa* was isolated only in a minority of four patients. Our data would thus suggest calculated treatment with glycopeptides or other antibiotics with activity against CNS and* Enterococcus* species.

Most of the isolated bacterial pathogens are part of the flora of gut and skin. This suggests a potentially crucial role of translocation through gastrointestinal mucosa due to mucositis on one hand and catheter-related infections on the other hand. In patients who succumbed to the initial sepsis, the time elapsed since the last replacement of central venous lines was significantly longer. Consistent with this observation, indwelling central line removal has previously been associated with better outcome [10]. Consequently, strategies for preventing bacterial translocation and strict removal or replacement of central venous lines after ICU admission should be advocated.

Notably, all patients who tested positive for active CMV infection deceased during their ICU stay. It remains unclear whether this active replication is only a marker of disease severity or plays a causative role in worsening prognosis either due to immunomodulation by or virulence of the virus itself. Controlled studies are needed to determine whether consequent prophylaxis or treatment may improve the course of illness in this subset of patients.

Two subgroups of patients had a particularly poor outcome: all patients after stem cell transplantation and all patients with a fungal infection on admission died in the course of their ICU stay. These observations are in line with previous publications that reported a very poor prognosis of patients after stem cell transplantation or neutropenic fungal infection in the ICU [8, 26].

In our cohort, patients who survived the sepsis received fewer blood transfusions. It is impossible to differentiate whether the higher number of transfusions is an indicator of a more severe disease, or if transfusions account for a worse prognosis. Problems with transfusion-associated lung injury and transfusion-associated inflammation are well known, and recent studies showed the benefit of restrictive transfusion strategies in different populations of ICU-patients [27, 28].

The main limitations of this study are its retrospective character and the fact that it is a single center study. Therefore, it is only hypothesis-generating and underscores the urgent need for prospective studies to further identify successful interventions in this subset of patients.

## 5. Conclusions

In patients with neutropenic sepsis, prognosis of the initial insult is determined mainly by severity of multiple organ failure. A significant proportion of patients who overcome the initial sepsis die in the further course of their ICU stay. In particular, patients with remaining organ dysfunction are at increased risk. Microbiological findings imply that all measures should be undertaken to preserve integrity of the gastrointestinal mucosa, for example, by early enteral feeding and central venous access should be replaced or removed following ICU admission. Adding antimicrobials with activity against Gram-positive cocci resistant to beta-lactam antibiotics early in the course of disease may have a significant impact on outcome.

## Figures and Tables

**Table 1:** Characteristics of patients who manage to overcome the initial sepsis compared to those who decease in the initial septic episode. Values are displayed as mean ± standard deviation; *∗*/*∗∗*/*∗∗∗* indicate level of significance.

Parameter	Initial sepsis, survived (*n* = 63)	Initial sepsis, deceased (*n* = 39)	*p* value	Odds ratio
Male	65,1% (*n* = 41)	69,2% (*n* = 27)	0,666	1,207
Female	34,9% (*n* = 22)	30,8% (*n* = 12)	0,666	0,828
Age	55,1 ± 14,9 (*n* = 63)	54,6 ± 11,9 (*n* = 39)	0,613	0,997
Days in the ICU	16,9 ± 17,0 (*n* = 63)	8,5 ± 7,6 (*n* = 39)	0,020	0,944^*∗∗*^
Duration prior to leukopenia	10,8 ± 16,3 (*n* = 52)	17,8 ± 18,5 (*n* = 36)	0,012	1,024
Respiratory frequency	26,1 ± 7,6 (*n* = 63)	29,5 ± 8,0 (*n* = 39)	0,032	1,058^*∗*^
First Lactate (mg/dL)	23,2 ± 19,3 (*n* = 63)	36,4 ± 37,8 (*n* = 39)	0,237	1,017^*∗*^
APACHE II on admission	22,8 ± 8,4 (*n* = 62)	29,0 ± 11,2 (*n* = 39)	0,003	1,069^*∗∗*^
SOFA on admission	7,3 ± 4,8 (*n* = 62)	10,1 ± 5,4 (*n* = 39)	0,011	1,115^*∗*^
Intubation	55,6% (*n* = 35)	89,7% (*n* = 35)	0,000	7,000^*∗*^
Lowest oxygenation index	112,8 ± 80,2 (*n* = 44)	81,4 ± 44,4 (*n* = 34)	0,035	0,99
Vasopressors in the first 24 h	63,5% (*n* = 40)	61,5% (*n* = 24)	0,843	0,92
Maximal noradrenaline dose (mg/h)	2,9 ± 3,2 (*n* = 45)	5,7 ± 3,7 (*n* = 36)	0,000	1,280^*∗∗*^
Fluid administration in the first 24 h (mL)	6710,8 ± 2527,0 (*n* = 63)	8960,5 ± 4790,8 (*n* = 39)	0,020	1,000^*∗∗*^
MAP first 24 h	56,1 ± 14,4 (*n* = 47)	55,1 ± 11,7 (*n* = 31)	0,617	0,994
Liver failure on admission	42,9% (*n* = 27)	66,7% (*n* = 26)	0,019	2,667^*∗*^
Lowest bilirubin	1,4 ± 1,7 (*n* = 63)	2,4 ± 2,8 (*n* = 39)	0,023	1,249^*∗*^
Lowest INR	1,2 ± 0,17 (*n* = 73)	1,4 ± 0,2 (*n* = 39)	0,000	49,677^*∗∗∗*^
Lowest urine output (mL/24 h)	1303,5 ± 1068,6 (*n* = 63)	540,1 ± 693,0 (*n* = 39)	0,000	0,999^*∗∗∗*^
Kidney failure	52,4% (*n* = 33)	79,5% (*n* = 31)	0,006	3,523^*∗∗*^
Renal replacement therapy	33,3% (*n* = 21)	71,8% (*n* = 28)	0,000	5,091^*∗∗∗*^
GCS on admission	12,7 ± 3,9 (*n* = 63)	13,1 ± 3,3 (*n* = 39)	0,876	1,031
GCS highest	14,6 ± 1,3 (*n* = 63)	13,2 ± 3,3 (*n* = 39)	0,002	0,735^*∗*^
Erythrocyte concentrates (units/day)	0,6 ± 1,1 (*n* = 63)	1,1 ± 0,9 (*n* = 39)	0,000	1,752^*∗*^
Platelet concentrates (units/day)	0,6 ± 0,6 (*n* = 63)	1,0 ± 0,6 (*n* = 39)	0,000	2,773^*∗∗*^
Fresh frozen plasma (units/day)	0,5 ± 0,8 (*n* = 63)	2,2 ± 2,8 (*n* = 39)	0,000	2,763^*∗∗∗*^
CRP highest first 10 days (mg/dL)	30,6 ± 9,9 (*n* = 63)	34,8 ± 16,8 (*n* = 39)	0,214	1,026
CRP lowest (mg/dL)	10,2 ± 8,7 (*n* = 63)	19,1 ± 10,8 (*n* = 39)	0,000	1,100^*∗∗∗*^
Persistent leukopenia	36,5% (*n* = 23)	82,1% (*n* = 32)	0,000	7,950^*∗∗∗*^

**Table 2:** Comparison of patients in the recovery phase after overcoming the initial sepsis. Values are displayed as mean ± standard deviation; *∗*/*∗∗* indicate level of significance.

Parameter	Recovery phase, survived (*n* = 46)	Recovery phase, deceased (*n* = 17)	*p* value	Odds ratio
Male	65,2% (*n* = 30)	64,7% (*n* = 11)	0,970	0,978
Female	34,8% (*n* = 16)	35,3% (*n* = 6)	0,970	1,023
Age	57,0 ± 13,9 (*n* = 46)	50,1 ± 16,7 (*n* = 17)	0,152	0,969
Days in the ICU	13,3 ± 13,5 (*n* = 46)	26,7 ± 21,6 (*n* = 17)	0,004	1,047^*∗*^
Duration prior to leukopenia	9,0 ± 15,8 (*n* = 46)	16,2 ± 17,3 (*n* = 17)	0,086	1,024
Respiratory frequency	25,7 ± 7,8 (*n* = 46)	27,4 ± 7,1 (*n* = 17)	0,243	1,031
First lactate (mg/dL)	21,0 ± 15,6 (*n* = 46)	29,0 ± 26,5 (*n* = 17)	0,368	1,02
APACHE II on admission	22,3 ± 7,8 (*n* = 45)	24,0 ± 9,9 (*n* = 17)	0,837	1,025
SOFA on admission	7,0 ± 4,7 (*n* = 45)	8,1 ± 5,2 (*n* = 17)	0,462	1,045
Intubation	39,1% (*n* = 18)	100% (*n* = 17)	0,000	53,919^*∗∗*^
Lowest oxygenation index	126,7 ± 94,2 (*n* = 27)	90,8 ± 45,3 (*n* = 17)	0,185	0,991
Tracheotomy	21,7% (*n* = 10)	64,7% (*n* = 11)	0,001	6,600^*∗∗*^
Lung failure after initial crisis	45,7% (*n* = 21)	94,1% (*n* = 16)	0,001	19,048^*∗∗*^
Vasopressors in the first 24 h	56,5% (*n* = 26)	82,4% (*n* = 14)	0,059	3,59
Maximal Noradrenaline dose (mg/h)	1,9 ± 1,6 (*n* = 28)	4,7 ± 4,2 (*n* = 17)	0,009	1,466^*∗*^
Fluid administration in the first 24 h (mL)	6670,7 ± 2375,0 (*n* = 46)	6819,5 ± 2977,2 (*n* = 17)	1,000	1
MAP first 24 h	56,0 ± 15,2 (*n* = 35)	56,6 ± 12,0 (*n* = 12)	0,643	1,003
Circulatory failure after initial crisis	4,3% (*n* = 2)	23,5% (*n* = 4)	0,021	6,769^*∗*^
Liver failure on admission	43,5% (*n* = 20)	41,2% (*n* = 7)	0,870	0,91
Lowest bilirubin	1,3 ± 1,2 (*n* = 46)	1,8 ± 2,6 (*n* = 17)	0,963	1,185
Lowest INR	1,2 ± 0,2 (*n* = 46)	1,2 ± 0,2 (*n* = 17)	0,871	0,792
Liver failure after initial crisis	37% (*n* = 17)	58,8% (*n* = 10)	0,120	2,437
Lowest urine output (mL/24 h)	1548,5 ± 1089,2 (*n* = 46)	640,5 ± 669,4 (*n* = 17)	0,001	0,999^*∗∗*^
Kidney failure	41,3% (*n* = 19)	82,4% (*n* = 14)	0,004	6,632^*∗∗*^
Renal replacement therapy	21,7% (*n* = 10)	64,7% (*n* = 11)	0,001	6,600^*∗∗*^
GCS on admission	12,7 ± 4,1 (*n* = 46)	12,8 ± 3,4 (*n* = 17)	0,871	1,005
GCS highest	15,0 ± 0,1 (*n* = 46)	13,7 ± 2,4 (*n* = 17)	0,000	0,070^*∗*^
Erythrocyte concentrates (units/day)	0,6 ± 1,2 (*n* = 46)	0,7 ± 0,5 (*n* = 17)	0,003	1,111
Platelet concentrates (units/day)	0,5 ± 0,7 (*n* = 46)	0,8 ± 0,5 (*n* = 17)	0,008	2,002
Fresh frozen plasma (units/day)	0,4 ± 0,9 (*n* = 46)	0,7 ± 0,6 (*n* = 17)	0,000	1,567
CRP highest in the first 10 days (mg/dL)	28,9 ± 8,8 (*n* = 46)	35,3 ± 11,5 (*n* = 17)	0,068	1,073^*∗*^
CRP lowest (mg/dL)	8,2 ± 6,5 (*n* = 46)	15,6 ± 11,4 (*n* = 17)	0,008	1,106^*∗∗*^
Persistent leukopenia	30,4% (*n* = 14)	52,9% (*n* = 9)	0,100	2,571

## List of Abbreviations

APACHE II: Acute Physiology and Chronic Health Evaluation Score II
CMV: Cytomegaly virus
CNS: Coagulase-negative staphylococci
ICU: Intensive care unit
EORTC: European Oganisation for Research and Treatment of Cancer
SD: Standard deviation
SIRS: Systemic inflammatory response syndrome
SOFA: Sequential organ failure assessment score
SSC: Surviving Sepsis Campaign.
